# Young people’s happiness in the context of negative life events and coping strategies: a latent profile and latent class analysis

**DOI:** 10.1186/s40359-023-01343-8

**Published:** 2023-10-14

**Authors:** Sema Polatcı, Ömer Lütfi Antalyalı, Ali Murat Alparslan, Seher Yastıoğlu

**Affiliations:** 1 Department of Business Administration, Tokat Gaziosmanpaşa University, Tokat, Turkey; 2 Department of Business Administration, Süleyman Demirel University, Isparta, Turkey; 3 Department of Public Relations and Publicity, Süleyman Demirel University, Isparta, Turkey; 4 Department of Business Administration, Burdur Mehmet Akif Ersoy University, Burdur, Turkey

**Keywords:** Negative life events, Coping strategies, Happiness, Latent profile analysis, Latent class analysis

## Abstract

Young people have to cope with many negative life events and stress factors to maintain their happiness. Although there are studies on how they benefit from different coping strategies and their results, there is no study that profiles/groups young people according to negative life events and coping styles. From this point of view, the study aims to determine different life events classes and stress coping profiles in young people, and to examine the differences in happiness among the new groups created according to the discovered classes and profiles. Participants consisted of 1093 young people (M = 21.08) from different state universities in Turkey. Latent profile analysis (LPA) was conducted, resulting in a 3 profile solution characterizing coping strategies: *Positive-oriented* (26.8%), *slightly positive-oriented* (50%), and *negative-oriented* (23.2%) *coping strategy profiles*. Latent class analysis (LCA) was conducted, resulting in a 2 class solution characterizing negative life events: *More negative* (40.1%) and *less negative* (59.9%) *life events classes.* For the purpose of the study, the profiles created with the latent profile analysis and the latent class analysis were considered together and the participants were divided into 6 groups. These groups differed significantly in terms of happiness. The group with the highest level of happiness is the group with positive-oriented coping strategies and less negative life conditions (*μ* = 4.35, *p* < .001), and the group with the lowest level of happiness is the group with negative-oriented coping strategies and more negative life conditions (*μ* = 3.48, *p* < .001). However, the findings indicated that a positive-oriented coping strategy profile (the profile that scored high on positive coping strategies and low on negative coping strategies) offers the most promising route to happiness whether experienced negative life events are less or more.

## Introduction

People may vary in positive and negative events they experience in their lives. Also, they may use more than one coping strategy when experiencing these life events [1, 2]. To investigate the link between the university students’ life conditions, coping strategies, and happiness levels we used latent profile analysis (LPA) and latent class analysis (LCA) which are contemporary techniques [3]. These two analyses allow researchers to discover profiles or groups of individuals, and create comprehensive results, rather than the traditional variable-centered approaches. In variable-centered approaches, the emphasis is on determining the relationships between variables and these relationships are assumed to be valid for all individuals. However, variable-centered analyses containing more than three interacting variables may be difficult to interpret and may be less suitable for making inferences about individuals [4]. It may cause statistical problems such as increases in the variance inflation factor and reduced statistical power [5]. Therefore, modeling interactions of interest is often impractical. In addition, the variable-centered approach may overlook the configuration of the characteristics within the individual [6]. In contrast, in person-centered approaches, the emphasis is on the whole individual. Individual subtypes that exhibit similar individual trait patterns are sought [7]. There is increasing interest in the person-centered approach, as the person-centered approach can provide a different perspective beyond the perspective put forward by the variable-centered approach (e.g. [8–10]). The person-centered approach describes how the traits under investigation are organized within individuals. Cataloging interactive effects as subtypes is practical in that it provides a concise and simple summary of complex relationships [11]. LCA and LPA can be used as statistical approaches to such situations [12]. These analyses, which can be considered as cluster analysis family, suggest a model-based approach to traditional clustering methods and have some advantages over traditional methods. A probabilistic model-based approach is more flexible [13], accounts for measurement errors [14], and is suitable for longitudinal studies [15]. In continuous data, we used LPA to identify the coping profiles of young people among different coping strategies. In categorical data, we used LCA to identify the life conditions of young people among different life events. These analyses are applied to identify distinct heterogeneity in students and illuminate homogenous subgroups to make the comparison of happiness levels. One of the important features that distinguishes this study from others is that this study aims to create meaningful subgroups, compare them with each other, and reveal the significant differences after the latent profile and latent class analyses.

Individuals seek a happy life and they think that they deserve it [16]. So many policymakers aim at greater happiness for a greater number. This pursuit of happiness calls for an understanding of the conditions for happiness and for that reason, the subject has received much attention [17]. In the past, it was believed that the happiness levels of people were stable [18, 19]. For example, in the set point theory (see [20]), it was argued that the happiness level of individuals is largely biologically driven and there are small fluctuations around this level [21]. Then, happiness was tied to material resources (such as money, house, car, vacation… etc.), and the positive effects of these factors on happiness were investigated. However, it has been seen that simple cognitive and behavioral strategies that individuals can apply in their daily lives are a more reliable way to reach happiness [22]. From this point of view, in recent years, happiness research has gained a different dimension, and it has been determined that strategies that can be applied by everyone and that will not cause major changes in their current life situations are shortcuts that lead to happiness. For example, Eryılmaz [23–25] also discussed the strategies to increase happiness in five dimensions: responding positively to the environment, getting positive reactions from the environment, performing mental control, satisfying desires, and fulfilling the requirements of religious belief. In 2017, Eryılmaz [26] developed a scale about happiness increasing strategies for adults.

Although there are many studies in the literature examining the relationship between life events and happiness [27–30] or coping strategies and happiness [24, 25, 31–35], there are still some unexplained gaps in the way these coping strategies used together and life events effect coping strategies of individuals.

This study focused on young people while searching for the relationship between life events, coping strategies, and happiness. According to Arnett [36], one of the most important duties of young people is identity exploration. He claims that the process of identity development includes many attempts and decisions made in various areas of life. This especially stressful process [37] makes young people more stressed and unhappy. The aim of this study is to group young people according to negative life events experienced and coping strategies used and then compare the happiness levels of these groups.

### Negative life events and happiness

Armstrong and his colleagues [38] define negative life events as “change events that precipitate movement from one set of living conditions to another”. The life transitions resulting from such events pose significant adaptational challenges that can strain people’s ability to cope to the point of clinical distress, manifest for instance in symptoms of stress, anxiety, and depression [38]. Indeed, experiencing these negative events can impede coping efficacy for additional events, increasing vulnerability to and even the likelihood of further negative events [39]. These negative experiences not only increase the stress level of the individual but also damage the well-being of individuals as they decrease positive emotions and increase negative emotions [40]. Even negative life events are one of the most robust predictors of poor well-being [41]. When these critical life events cannot be coped with or the conditions cannot be adapted, the negative effects on well-being can continue for a long time [42].

The positive and negative events that individuals encounter in their lives affect their happiness [43]. Luhmann et al. [29], made a meta-analysis examining the effects of life-long events on happiness and listed life events related to work and family that affect happiness. Clark and Oswald [28] focused on the effects of life events on happiness from a mathematical perspective and developed a statistical model explaining the interaction. According to this model, the psychological costs of losing a job are higher than a pure drop in income. On the other hand, getting married makes people happy as they have an extra £70,000 of income per annum.

According to studies, negative life events have a greater impact on happiness than positive life events [27, 44]. For this reason, this research focused on negative life events. The negative events experienced a negative impact on the healthy development of the individual, causing psychological problems [40, 45], depression [46, 47], and anxiety [40, 48], while harming the mental health of the individual [49].

It is known that negative life events are one of the most important factors that affect happiness negatively [41, 50]. In addition, studies show that negative life events influence happiness not only after the event, but the average level of happiness fails to return to its original level for the rest of the individual's life. According to this effect, which is a phenomenon known as the “scarring effect”, the traces of negative life events above the happiness level of the individual remain for life [51]. While it is known that negative life events affect happiness negatively, it has been determined that this situation is not experienced in the same way in all individuals. Some individuals can get out of negative life events with the least damage with the resources they have or the strategies they have developed. It has even been observed that some individuals become stronger after these events and develop resilience afterward. For example, Grant et al. [52], state that negative events encountered in childhood have a positive effect on the resilience of the individual in older ages. Contrary to all these studies, Valiant [53] argues that happiness is not affected by the cognitive evaluation of life events.

When individuals are faced with a negative life event, they can manage it. However, when faced with two or more negative life events in five-years, their well-being seems to decrease significantly [54]. Therefore, it would not be wrong to expect that the more negative life events experienced compared to the individual average level, the greater the downward change in the level of well-being [55].

People are faced with negative life events both from themselves and from family, such as exposure to serious illness, death of a family member/relative, financial difficulties, legal problems [56], conflicts with parents [57], academic stress [58], parental divorce [59], family problems, etc. [60]. Therefore, the negative life events experienced by the individual can be examined in two groups; related to himself and related to his/her family. Studies have shown that negative life events related to family have a negative effect on happiness as well as negative life events related to himself [61].

### Coping strategies and happiness

Coping is a multidimensional construct that encompasses cognitive, emotional and behavioral regulatory processes to manage specific demands encountered during a stressful situation [62]. When faced with difficulties, individuals resort to coping strategies.

Many coping strategies are mentioned in the literature. Skinner et al. [63] listed 400 different coping strategies and made analyses to reduce them. As the potential number of specific coping responses is infinite, it is imperative to categorize them in one way or another. In fact, several authors have proposed coping measures with considerably more dimensions. For example, COPE offered 13 ways [64], SACS offered 9 [65], Folkman and Lazarus offered 7 [66], and the most extreme number is offered by McCrae [67] which identifies 28 ways of coping. So, it is obvious there is no agreement on the number of dimensions which is adequate to describe coping behavior [68]. While studies use different coping strategies, coping strategies that are predominantly used also emerged. In this study, according to the literature review, seven of the most commonly used coping strategies were used. So this study combined the coping strategies of Folkman and Lazarus [66] with Zuckerman and Gagne [69], Garnefski and his colleagues [70]. These are submissive strategy, optimistic strategy, seeking social support strategy, positive reappraisal strategy, blaming others strategy, avoidance strategy, and self-punishment strategy [1, 63, 64, 71–73]. The *submissive strategy* is the strategy in which an individual accepts to experience stress-related negativities by exhibiting a fatalistic attitude [74, 75]. The *optimistic strategy* is the exhibition of an optimistic attitude in which the individual controls himself regarding the stressful situation and approaches the events reasonably [75]. The *seeking social support strategy* argues that it is useful to receive help from others to reveal the reason for a stressful situation and to deal with it [76]. The *positive reappraisal strategy* refers to thoughts of creating a positive meaning to the event in terms of personal growth [77]. People, who use positive reappraisal strategies, may more easily tolerate or master negative life experiences [78]. In the *blaming others strategy* individual puts the blame for what he has experienced on the environment or other individuals [77, 79]. The *avoidance strategy* represents behaviors that orient the individual away from the problems [69]. The *self-punishment strategy* is the strategy of putting oneself at the source of the problem, self-blaming, being mentally preoccupied with the problem, suppressing the ability to struggle, and pessimism [69].

There are several factors that affect happiness, but the coping strategies against the difficulties encountered have a significant impact on happiness [31, 32, 80]. Some types of coping strategies are more effective than others in maintaining happiness. For example, facing the negative events will better maintain or improve the level of psychological well-being compared to avoidance coping or avoidance of the problem. Individuals who can provide a suitable response to the negative events faced, even though he is in a state of stress will positively affect the level of psychological well-being of the individual [33]. Some research was conducted on Turkish youth, and they revealed interesting results. In coping with negativities; solving the problem causing unhappiness, avoiding to use any strategy, increasing physical well-being, distancing themselves from the sources of happiness, receiving and giving social support, and achieving mental control have been observed [24, 25]. Two coping strategies, seeking social support and avoidance strategies, are important in maintaining happiness in the face of difficulties [35].

### The current study

Differences in conceptualization have led to several ways of classifying coping strategies. The most widely used dimensions of coping are problem-focused or emotion-focused coping, primary or secondary control coping, and engagement (approach) versus disengagement (avoidance) coping [81, 82]. However, criticism of these dimensions is also widespread, because dimensions are overly broad and place many disparate types of coping into these two general categories (e.g. [82, 83]). One of the alternative conceptualizations, the one utilized in the present study, was recommended by Sawyer et al. [84]. He defines the tendency to react to or deal with problems in a constructive, direct, positive manner as a positive coping strategy, and the tendency to react to or deal with problems or issues in an avoidant or unconstructive manner as a negative coping strategy. According to Sawyer et al. [84] we called optimistic, seeking social support, and positive reappraisal strategy as *positive coping strategies*; submissive, blaming others, avoidance, and self-punishment strategy as *negative coping strategies* in this study*.*

It has been determined that life events have a positive effect on the development of coping strategies over time [85], and those who experience less negative life events are weaker in developing and using coping strategies [26]. It is thought that experiencing negative life events has such an effect as it develops the cognitive and behavioral resources that the individual needs to develop a coping strategy [86]. Studies are showing that coping strategies used are effective in happiness [87].

Coping strategies have been investigated in some studies as mediating variables in the effects of negative life events on happiness in adolescents and adults [50, 88–91]. In some studies, coping strategies were considered as moderators between life events and psychological consequences [92, 93]. Although it is generally accepted that exposure to negative life events will lead adolescents to mental health problems, it is noteworthy that some adolescents are resistant even under these adverse conditions [94]. These adolescents are able to overcome negative life events without being affected psychologically. Therefore, these findings point to the need for more research on the link between negative life events, coping strategies, and happiness.

Individuals may be exposed to different negative life events (easy or difficult). Same way; they can develop different coping strategies unique to themselves in coping with stress or negative life events. It has been demonstrated by previous studies that experiencing negative life events causes the implementation of different coping strategies [94] and that life events (e.g. [29, 95, 96] and coping strategies have different effects on individuals' well-being levels (e.g. [97]). For example, avoidance coping strategies are associated with more negative life events and psychological problems [98]; and blaming others and self-punishment coping strategies are associated with more depression [99]. Cognitive strategies such as seeking social support and positive reappraisal can reduce the negative impact of negative life events on psychological health and provide greater satisfaction with life despite negative life events [100]. As can be seen, positive or negative life experiences and reactions can differ. However, it is predicted that individuals may have different profiles that are consistent within themselves according to different coping strategies. Again, it is predicted that these differences and similarities can be grouped with negative life events and happiness levels may differ according to these groups.

In the light of these information, this study has two aims. The first aim of the study is to identify and describe the coping strategy profiles and negative life event classes in a sample of university students (young people) using latent profile analysis and latent class analysis from an exploratory perspective. The second aim of the study is to examine the differences in the happiness levels of young people through new profiles and classes formed in terms of negative life events and coping strategies. This will enable access to more in-depth details of the findings.

Based on the aim of the study, the happiness levels of university students were examined according to their strategies to cope with stress and the negative life events they encountered in the last 1 year. While 3 positive (optimistic, seeking social support, and positive reappraisal strategy) and 4 negative (submissive, blaming others, avoidance, and self-punishment strategy) coping strategies were taken as the basis for coping with stress, 2 different dimensions (family events, individual life events) were used for negative life events. We used LPA and LCA to discover profiles or groups of individuals and create comprehensive results, rather than the traditional variable-centered approaches, which examine relations among variables [101]. We used LPA to identify the coping profiles of students among different coping strategies, and we used LCA to identify the life conditions of students among different life events.

## Method

### Participants and procedure

The data from the current study was collected from different state universities in Turkey. The sample of the study consists of 1093 undergraduate and graduate students. A convenience sampling procedure was used to identify participants. The suitability of the sample size was examined through the reference values suggested in the literature, in the context of the statistical analyses carried out in the study. The sample size of a minimum of 500 participants is considered sufficient to determine the correct number of latent profiles when performing profile analysis [102]. In the multiple regression analysis performed with seven dependent variables, it is stated that the minimum number of samples needed to obtain a power level of 0.80 should be 682 [103]. In addition, post hoc power analysis was performed in G*Power v3.1.9.7 software, by taking the results of one of the multiple regression analyses in the findings section of this study as a reference. Accordingly, the test power was calculated as 1.0 for 7 predictors, 1093 total sample size, α err probe 0.05, and effect size f^2^ 0.32. According to the available evidence, it can be said that the sample size of the study is sufficient for the analyses to be performed.

A quantitative research design [104] was used in the study because the research conducted for the study was based on numerical data and the research variables reported a degree or amount. Data were collected from the field using the online survey technique. The questionnaire form links were sent to the participants via Google Forms. All participants filled out the questionnaires voluntarily. On the first page of the online questionnaire, the participants were informed about the purpose of the study, and that they could leave the study at any time and for any reason. In addition, in this section, information was given on the confidentiality of participant information and the anonymization of data. Before the data collection process, Institutional Review Board permission for research was obtained from the University’s Ethical Committee in 2021.

Of the 1093 undergraduate and graduate students in the sample, 70.7% were (*N* = 773) women and 93.7% were undergraduate students. Differences in the sample distribution were not consciously chosen. As stated before, the link to the questionnaire was sent to the students via online communication channels (e.g., message, mail) and participation in the research was made voluntarily. However, the number of undergraduate students was higher than the number of graduate students. Their ages ranged from 18 to 30 years (M = 21.08, sd = 2.348). Approximately half of the sample (49.2%) were in the 18–20 age range; 38.7% were 21–23, 7.4% were 24–26, and 4.7% were in the 27–30 age range.

### Measures

#### Negative life events inventory (NLEI)

Participants’ negative life events experiences were measured 20-item checklist of negative life events based on previous inventories [60, 105, 106]. With each item, participants are asked to indicate whether the event had occurred during the previous year, using a dichotomous (yes–no) response scale. The inventory includes 11 events that occurred to family members and 9 events that occurred directly to the individual. A higher score reflects more negative life events adding scoring response 0 = No and 1 = Yes. The internal consistency coefficient (Cronbach’s α) of the total scale was calculated as 0.67-0.71. A high score on the test, that is, more yes answers means that more negative life events are experienced, or vice versa. In this study, a 20-item inventory, which includes 11 events that occurred to family members and 9 events that occurred directly to the individual inventory, was used to measure NLEI. The inventory was adapted to Turkish by the researchers following the scale validation procedures. In the adaptation process, first of all, the translation back translation process from English to Turkish was operated by 3 language experts of the measurement tool. Thus, it has been tried to prevent possible loss of meaning in the translation. Then, the suitability of the translation, the clarity of the items, and the representation of the structure were evaluated by 2 academicians working in the field of psychology, and evidence was obtained for the content validity of the measurement tool. In this study, Cronbach’s alpha for total NLEI is 0.68.

#### Coping strategies measurement tools

We used 7 different coping strategies to determine the coping styles of the university students. To measure submissive style, optimistic style, and seeking social support strategies “Ways of Coping Inventory” developed by Folkman and Lazarus [66] and adapted into Turkish by Şahin and Durak [74] was used. The original inventory has 66 items, however, the inventory was shortened while adapting to Turkish culture which has 33 items and 5 subscales. We used 3 sub-dimensions of this inventory so have 6 items to measure submissive strategy, 5 items to measure optimistic strategy, and 4 items to measure seeking social support strategy. A 4-point Likert-type rating was used. In this study, confirmatory factor analysis (CFA) was performed for each subscale and the results were at an acceptable level (χ2 = 42.6, CFI = 0.97; TLI = 0.92, RMSEA = 0.074, SRMR = 0.031; χ2 = 3.3, CFI = 0.99; TLI = 0.99, RMSEA = 0.024, SRMR = 0.008; χ2 = 0.38, CFI = 1.00; TLI = 1.00, RMSEA = 0.000, SRMR = 0.002). The internal consistency reliability coefficient of the subscales ranged from 0.63-0.76.

To determine the avoidance and self-punishment strategies of the participants the Revised Cope Inventory developed by Zuckerman and Gagne [69] adapted into Turkish by Dicle and Ersanlı [107] was used. The scale has a 4-Likert type rating. Each of the subscales used to measure responses to coping with stress consists of 6 items. The internal consistency reliability coefficient (α) of the dimensions of the scale was 0.98 [107]. In this study; as a result of the CFA performed with each subscale, avoidance, and self-punishment subscales’ one-dimensional structure was confirmed, respectively (χ2 = 14.4, CFI = 0.99; TLI = 0.98, RMSEA = 0.035, SRMR = 0.017; χ2 = 14.7, CFI = 0.99; TLI = 0.99, RMSEA = 0.037, SRMR = 0.012). The internal consistency reliability coefficient of the subscales was 0.69 and 0.86, respectively.

Positive reappraisal and blaming others strategies were measured with the Cognitive Emotion Regulation Questionnaire (CERQ) which was developed by Garnefski and his colleagues [108]. In this study, 2 dimensions of the scale were used and each dimension consisted of 4 items. The scale was adapted into Turkish by Ataman [109]. A 5-point Likert type was used in the scale. In the adaptation study of Ataman et al. [109], the internal consistency coefficient of the positive reappraisal subscale was calculated as 0.79, and blaming others subscale was calculated as 0.83. In this study, a result of confirmatory factor analysis for the positive reappraisal subscale’s and blaming others subscale’s one-dimensional structure consisting of 4 items was supported as in the original scale (χ2 = 1.71, CFI = 1.00; TLI = 0.99, RMSEA = 0.025, SRMR = 0.004; χ2 = 0.030, CFI = 1.00; TLI = 1.00, RMSEA = 0.000, SRMR = 0.000)). Cronbach’s Alpha coefficient of the subscales was determined as 0.86 and 0.82.

Although the CFA results performed separately for 7 subscales representing the top-down strategies give acceptable model values, in this study, CFA using the Jamovi [110] package program and exploratory structural equation modeling (ESEM) using the package program's module SEMLj [111] to test the integrity of the model. In the analysis made with the classical CFA approach, a single measurement model including 7 scales was tested. As a result of the analysis, the RMSEA value was determined as 0.0574 at a 90% confidence interval (CI = 0.055; 0.0510). ESEM [112, 113], an advanced method that combines the functions of exploratory factor analysis (EFA) and CFA in a single analytical framework, allows cross-loading between items and factors simultaneously, as well as harmonization. It calculates goodness indexes, allows estimation of error terms, and allows for invariance testing [112]. As a result of ESEM made on the model with 7 scales, the RMSEA value was calculated as 0.054 in 95% confidence Intervals (CI = 0.051; 0.057). RMSEA values obtained as a result of both DFA and ESEM are among the accepted threshold values. Therefore, it can be said that coping strategies scales are suitable for the integrity of the model and support the theoretical assumptions.

#### Happiness scale (HS)

Students’ perceptions of general life happiness were measured with the Happiness Scale developed by Demirci and Ekşi [114]. It has a one-dimensional structure consisting of 6 items and is rated on a 5-point scale. The Cronbach’s α coefficient of the original scale was found to be 0.83 and the test–retest reliability coefficient of 0.73 [114]. In this study, a result of confirmatory factor analysis for the Happiness Scale’s one-dimensional structure was found as valid and reliable (χ2 = 17.6, CFI = 0.99; TLI = 0.99, RMSEA = 0.042, SRMR = 0.011). Cronbach’s alpha was determined as 0.88.

### Statistical analysis

Data distribution statistics, reliability coefficients, test of normality analyses, and confirmatory factor analyses were all done with the Jamovi [110] package program. Before starting the analysis, the collected data were examined in terms of missing or extreme values and made ready for analysis by performing the necessary procedures. After the analysis, skewness and kurtosis values ​​were checked [115]. We performed LPA and LCA in the R v4.2.0 [116] program with its RStudio v2022.02.3.492 [117] interface, to reach our research aim. The mclust v5.4.9 package [118–120] was used for LPA, and the poLCA v1.6.0.1 [121] package was used for LCA. Graphics were drawn by the ggplot2 v3.3.6 package [122]. For ANOVA and regression analysis, the R-based Jamovi v2.2.5 [110, 117] was used.

Finite gaussian mixture models were used to classify individuals according to their strategies for coping with stress and negative life events. While continuous data is used as input in latent profile analysis (LPA), dichotomous or polytomous data is used in latent class analysis (LCA), which are from the cluster analysis family [123]. To classify individuals according to coping strategies, LPA was carried out using seven strategies that measure coping strategies. With this analysis, it was predicted that profiles of coping strategies will emerge. On the other hand, to classify individuals according to negative life events, LCA was carried out by using the items of the negative life events scale, which were answered as "yes—no". Here, too, it is predicted that different classes will emerge, which differ from each other according to the negative life events of the individuals.

In addition, Harman's one-factor (or single-factor), one of the most frequently used methods, was used to evaluate the common method bias (variance) before analyzing the main findings of the study. In order to perform the test, all the variables of the research were included in the factor analysis, all variables were loaded on a single factor, and the analysis was carried out by limiting it to principal components analysis and unrotated factor solution [124]. As a result of the analysis, according to the findings obtained from the data of this study, the variance of the newly added common latent factor was determined as 13.5%. Since the common latent factor explains less than 50% of the variance, it can be said that there is no common method bias [125].

## Results

### Latent profiles in coping strategies

LPA was conducted to discover latent profiles in participants according to coping strategies with stress. For the EM (expectation–maximization) initial algorithm in LPA; all parameters (variance–covariance) were released and MBHAC (model-based hierarchical agglomerative clustering) method was used based on scaled SVD (singular value decomposition) [121]. As a result of the analysis carried out with seven coping strategies, BIC suggested 6 profiles (-14,647) at first rank, 8 profiles (-14,669) at second rank, and 3 profiles at third rank (-14,747). In addition to BIC, ICL, which penalizes entropy, suggested 6 profiles (-15,078) at first rank and 3 profiles (-15,111) at second rank. (Most modeling applications use this formula “df(log(n))-2(LL)” in BIC calculations. The smallest value represents the best model. For some theoretical reasons, BIC in mclust is calculated with the formula “2(LL)-df(log(n))”, and the highest value represents the best model [126]. The same is true as ICL is calculated based on BIC [127].

**Fig. 1:**
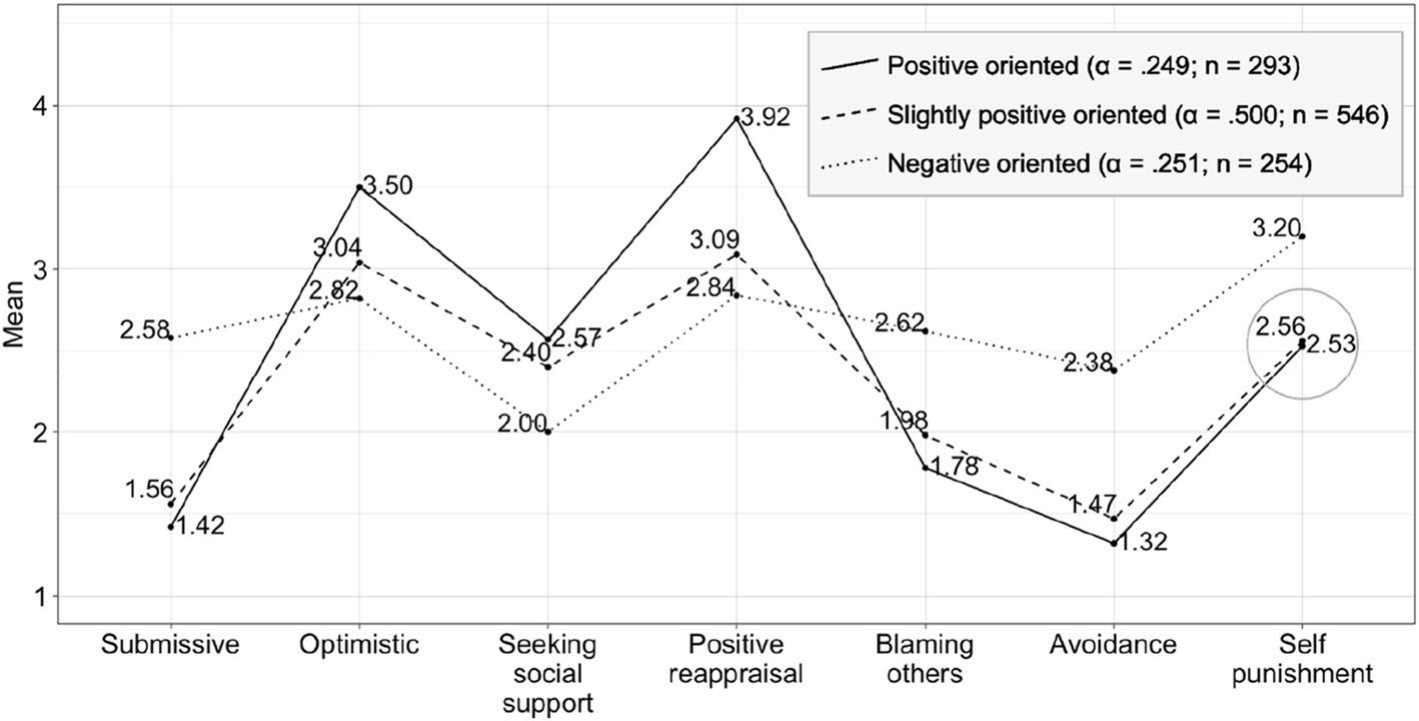
Latent profile analysis according to coping strategies α: Profile weights; n: Number of participants after assignment. Note: Averages are calculated on the basis of profiles after assignment

In order to compare the aforementioned models, it was investigated how much of the proportion of variability in the profiles was caused by the pattern and how much was caused by the level (see for details: [128–130]). To understand whether the level and pattern of scores of the participants can predict the profiles, the cpa function in the profileR package was used [131]. Pattern effect is close to each other in all three proposed models (R^2^_3-profile_ = 0.221, R^2^_6-profile_ = 0.255, R^2^_8-profile_ = 0.171). However, the level effect seems higher in the model with 3 profiles (R^2^_3-profile_ = 0.200, R^2^_6-profile_ = 0.051, R^2^_8-profile_ = 0.003). If an overall evaluation is made, the proportion of variability in profiles explained by the full model is higher in the 3-profile model than in the 6- and 8-profile models (R^2^_3-profile_ = 0.374, R^2^_6-profile_ = 0.267, R^2^_8-profile_ = 0.196). The findings increased the interest in the 3-profile model. In the literature, parsimony is recommended in the number of profiles [132] and in cases where there are similar profile structures, it was necessary to tend towards lower profile numbers [133]. There are similarities in profile structures in models with 6 and 8 profiles. In addition, the 3-profile model was theoretically more interpretable. As a result, it was decided to choose a 3 profile model. 3 profile model, was in varying volume, varying shape, and equal orientation (VVE) structure [121]. The Lo-Mendell-Rubin test (71.9) showed that 3 profiles should be preferred over 2 profiles (*p* < 0.01). APPA (average posterior probability assignment) findings in 3 profiles were over 0.8 (0.90 -0.82 -0.87) and normalized entropy was calculated as 0.70. Uncertainty levels of 72% of the participants were below 0.2 and 99% of them were below 0.5. Profiles obtained as a result of LPA were added to the data set as a new variable and the averages calculated on the basis of profiles were plotted graphically in Fig. 1.

As predicted, a strong profile of coping strategies emerged. Latent profile analysis identified 3 coping profiles: *positive-oriented profile (PO)* (the profile that scored high on positive coping strategies and low on negative coping strategies) (a = 0.249, *N* = 293), *slightly positive-oriented profile* (SPO) (the profile with average scores from positive and negative coping strategies) (a = 0.500, *N* = 546), and *negative-oriented profile* (NO) (the profile that scored high on negative coping strategies and low on positive coping strategies) (a = 0.251, *N* = 254).

For each strategy, comparisons were made according to profiles with a one-way analysis of variance test. As a result of the Games-Howell Post Hoc test, no difference was observed between the positive-oriented and slightly positive-oriented profiles only in terms of self-punishment, which were circled in Fig. 1 (*p* = 0.81). All other differences are significant (*p* < 0.01). It can be said that all profiles diverge quite well. For the validation of the profiles, the happiness levels between profiles were compared with a one-way analysis of variance and it was found that there was a significant difference (µ_PO_ = 4.30, µ_SPO_ = 3.88, µ_NO_ = 3.59, F_Welch_ = 53.3, *p* < 0.01). According to the Games-Howell Post Hoc test, all differences between profiles are significant (*p* < 0.01).

### Latent classes in negative life events

**Fig. 2:**
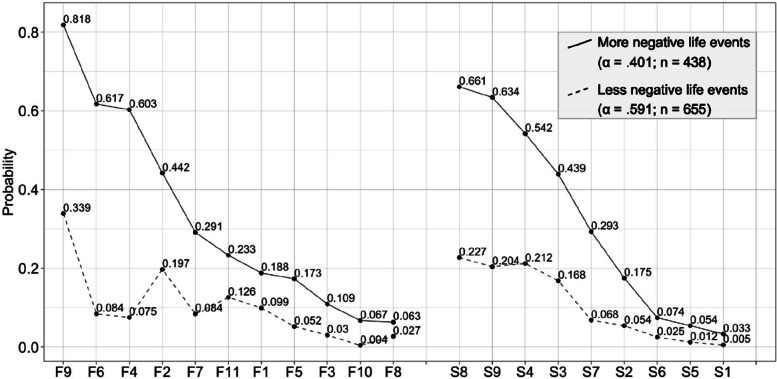
Latent class analysis according to negative life events F: Family items, S: Self items, α: Class weights n: Number of participants after assignment

LCA was performed with the items of the negative life events scale. The poLCA package, which leaves the covariates free, runs the analysis repeatedly to reach the global maximum and finds the best result. After 50 replications, BIC recommended 2 classes (BIC_1_ = 18,589, BIC_2_ = 17,655, BIC_3_ = 17,735) at first rank. The segregation in the 2-cluster structure is in line with the expectation. The Lo-Mendell-Rubin test (1031.7) showed that 2 profiles should be preferred over 1 profile (*p* < 0.01). APPA findings were above 0.8 (0.93—0.91) and normalized entropy was calculated as 0.73. Uncertainty levels of 84% of the participants are below 0.2, and 100% of them are below 0.5. The probability of giving a "yes" answer to each item on the basis of classes as a result of LCA is shown in Fig. 2.

For both items about family and about himself, the participants in one class are more likely to answer "yes" than the participants in the other class. The latent class analysis identified two classes according to the answers: *less negative life events class* (LNLE) (those who experienced fewer negative life events in the last 1 year) (a = 0.591, *n* = 655), and *more negative life events class* (MNLE) (those who experienced negative life events more frequently in the last 1 year) (a = 0.401, *n* = 438). For the validation of the classes, the happiness levels between the classes were compared with the independent samples t test and it was found that there was a significant difference (µ_LNLE_ = 4.06, µ_MNLE_ = 3.72, t_Welch_ = 6.34, *p* < 0.01).

### Comparison of happiness levels

As a result of LPA (3 profiles according to different coping strategies) and LCA (2 classes according to negative life events), 6 groups were formed. Among the participants with negative-oriented coping strategies (NO), 108 had less negative life events (LNLE), and 146 had more negative life events (MNLE). Among the participants with slightly positive-oriented coping strategies (SPO), 351 had less negative life events (LNLE), and 195 had more negative life events (MNLE). Finally, among the participants with positive-oriented coping strategies (PO), 196 had less negative life events (LNLE), and 97 had more negative life events (MNLE). To understand the differences between the groups, the effects of coping strategies on happiness were examined together. Regression analyses were performed separately on the basis of groups and the findings are presented in Table 1.

**Table 1:** The effect of coping strategies on happiness on the basis of groups

*Predictors*	*NO-LNLE*	*NO-MNLE*	*SPO-LNLE*	*SPO-MNLE*	*PO-LNLE*	*PO-MNLE*
*Submissive str*	-.216^*^	-.126	-.088	.055	-.090	-.114
*Optimistic str*	.047	.165	.273^***^	.426^***^	.218^*^	.155
*Seeking social support str*	.031	.254^**^	.123^*^	.151^*^	.147^*^	.108
*Positive reappraisal str*	.271^*^	.074	.159^***^	.071	.005	.199^*^
*Blaming others str*	-.008	.189^*^	-.033	-.155^*^	.024	.009
*Avoidance str*	.099	-.099	.003	-.022	-.104	.113
*Self-punishment str*	.103	-.001	-.133^*^	-.024	-.064	-.289^**^
*R*^*2*^	.156	.175	.191	.240	.131	.177
*N*	108	146	351	195	196	97
*F(df1,df2)*	(7,100)	(7,138)	(7,343)	(7,187)	(7,188)	(7,89)
*F*	2.64	4.17	11.60	8.42	4.04	2.73
*p*	< .001	< .001	< .001	< .001	< .001	.013

All estimates in the table are standardized estimates. F and p values are for overall model tests

*NO* Negative-oriented coping strategy profile, *SPO* Slightly positive-oriented coping strategy profile, *PO* Positive-oriented coping strategy profile

*LNLE* Less negative life events class, *MNLE* More negative life events class

^*^p < .05

^**^p < .01

^***^p < .001

Among the coping strategies, it was observed that the avoidance strategy had no significant effect on happiness in any group, while the coping strategies with the most significant effect in the groups were seeking social support strategy, positive appraisal strategy, and optimistic strategy. The coping strategies that stood out in all groups were different. For example, the happiness level of the participants in the NO-LNLE group was affected by submissive (β = -0.216, *p* < 0.05) and positive reappraisal (β = 0.271, *p* < 0.05) strategies. The happiness level of the participants in the PO-LNLE group was affected by optimistic (β = 0.218, *p* < 0.05) and seeking social support (β = 0.147, ***p*** < 0.05) strategies. In the NO-MNLE group, happiness was affected by seeking social support (β = 0.254, *p* < 0.01) and blaming others (β = 0.189, *p* < 0.05) strategies. As a result, it was concluded that the effects of coping strategies on happiness vary according to the latent profile and latent class of the individual. It can be said that there was a differentiation between the groups obtained as a result of LPA and LCA analyses, which is worth examining.

**Fig. 3:**
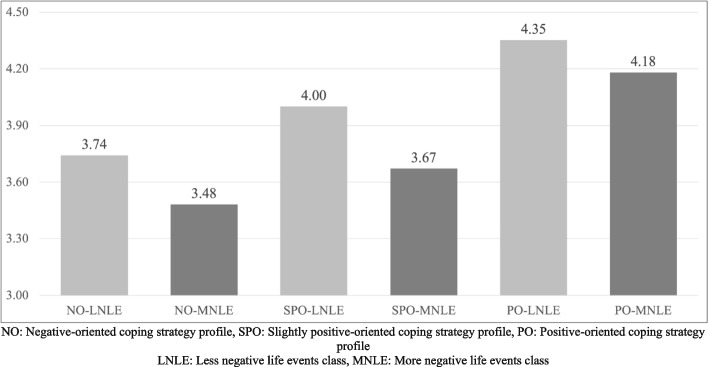
Happiness levels by groups NO: Negative-oriented coping strategy profile, SPO: Slightly positive-oriented coping strategy profile, PO: Positive-oriented coping strategy profile LNLE: Less negative life events class, MNLE: More negative life events class

After these examinations regarding the groups, we examined the change in happiness levels according to groups. The difference between the happiness levels of the 6 groups was tested with a one-way analysis of variance. In addition, the differences between all groups were examined with the Games Howell Post Hoc test. The findings are shown in Table 2, and the average happiness levels of the groups are plotted graphically in Fig. 3.

**Table 2:** Change in happiness levels by groups

*Groups*	*NO-LNLE*	*NO-MNLE*	*SPO-LNLE*	*SPO-MNLE*	*PO-LNLE*	*PO-MNLE*
	*Welch test F(5.384)* = *28.9, p* < *.001*					
*NO-LNLE (n* = *108)*	3.74(*.97*)	-.26*	.30**	**-.07**	.75**	.49**
*NO-MNLE (n* = *146)*	-.26	3.48(*1.01*)	.58**	**.20**	1.04**	.76**
*SPO-LNLE (n* = *351)*	.26	.52^**^	4.00(*.76*)	-.41**	.50**	.23*
*SPO-MNLE (n* = *195)*	-.07	.19	-.33^**^	3.67(*.81*)	.94**	.62**
*PO-LNLE (n* = *196)*	.61^**^	.87^**^	.35^**^	.68^**^	4.35(*.62*)	**-.23**
*PO-MNLE (n* = *97)*	.44^*^	.70^**^	.18	.51^**^	-.17	4.18(*.82*)

The happiness averages and standard deviations of the groups are located on the diagonal in the form of µ(sd)

The lower diagonal is the difference between the average happiness of the group in the row and the group in the column. The upper diagonal is Welch t tests Cohen d effect size

*NO* Negative-oriented coping strategy profile, *SPO* Slightly positive-oriented coping strategy profile, *PO* Positive-oriented coping strategy profile

*LNLE* Less negative life events class, *MNLE* More negative life events class

Those with a significant difference in the Games Howell Post Hoc test result are marked with*

^*^p < .01

^**^p < .001

When the averages of happiness were examined on the basis of groups, it was observed that the group with the highest happiness level was PO-LNLE (*μ* = 4.35), and the group with the lowest happiness level was NO-MNLE (*μ* = 3.48). The importance of coping strategies was understood when the change in happiness levels was examined according to the coping strategies by keeping the negative life conditions constant. Participants in less negative life events class and having positive-oriented coping strategies (PO-LNLE) were happier than the others (*p* < 0.001). In addition, among the more negative life events class, individuals with positive-oriented coping strategies (PO-MNLE) were happier than the others (*p* < 0.001). However, it should be noted that the slightly positive-oriented profile in both less and more negative life events classes was not sufficient to increase happiness compared to the negative-oriented coping strategy profiles (*p* > 0.05). The effect size values for the group differences were also calculated and given in the upper diagonal of Table 2.

The importance of coping strategies was better understood when the change in happiness levels according to more negative life events is examined by keeping the coping strategies constant. Happiness levels of those who had a negative-oriented profile (NO) did not change even if their negative life events were less or more (*p* > 0.05). On the other hand, negative life conditions affected the level of happiness in those with a slightly positive-oriented profile (SPO). Among these groups, it could be said that those with less negative life events class (LNLE) were happier than those with more negative life events class (MNLE) (*p* < 0.001). Happiness levels of those with a positive-oriented coping strategies profile (PO) were not affected by negative life conditions, as NO groups. The happiness levels of the participants in the PO groups were high and there was no significant difference according to whether the negative life events were more (*μ*_*PO-MNLE*_ = 4.18) or less (*μ*_*PO-LNLE*_ = 4.35) (*p* > 0.05).

## Discussion

It is important to determine the coping profiles of young people who are faced with different negative life events, and to reveal which coping strategies can be most compatible with which life conditions to increase the happiness of young people. Furthermore, identifying what type of coping strategy predicts adequate happiness level in conjunction with difficult life events can provide valuable information on developing the most adaptive strategy during youth. From this point of view, in this study, it was aimed to obtain meaningful and different groups related to different life events and stress coping strategies in young people by latent profile and latent class analyses and to examine the differences in happiness levels between these groups.

Individuals are classified according to the most suitable profiles. While doing this, the general fit of the model and probabilities are used [134]. The popularity of LPA and LCA is increasing. Because in continuous data LPA, and categorical data LCA can identify clusters across numerous factors [135]. We conducted LPA on the coping strategy preferences of the university students. And achieved 3 profiles of strategy combinations: positive-oriented, slightly positive-oriented, and negative-oriented. Our profiling and its effects on happiness have some similarities with coping strategy profiles in previous research [2, 63, 136, 137, 138]. For example, Kavčič et al. [136] classified the individuals who took high scores from positive coping strategies (active coping, positive reframing, planning, and acceptance) as engaged coping profiles and this profile reported the highest levels of well-being. We conducted LCA on the negative life events of students in the last 1 year. After LCA, we achieved 2 classes of life conditions: less negative life events and more negative life events. These profiles were similar to those found in previous studies [139, 140]. As a result of LPA and LCA, 6 groups were created, and the most crowded group was a slightly positive-oriented coping strategy-less negative life events group with 351 students.

Results indicated that positive-oriented coping strategy profiles (the profile that scored high on positive coping strategies: seeking social support strategy, positive reappraisal strategy, and optimistic strategy, and low on negative coping strategies: submissive strategy, blaming others strategy, avoidance strategy, and self-punishment strategy) have a significant effect on happiness in most of the groups. Many studies supporting these findings were found in the literature [100, 141, 142]. Seeking social support strategy stands out with affecting happiness in more groups than the other 2 positive coping strategies. Social support (e.g., support from family or close friends) is recognized as one of the most effective ways to cope with stressful events in daily life [143]. Therefore, it can affect the happiness levels of individuals positively by reducing the effects of negative life experiences and other stressors [141]. It is suggested that the presence of social support protects the individual from the negative consequences of stress (for example, anxiety or depressive thoughts) when faced with threatening life events [144]. In a study conducted on adolescents, it was found that the strategy of seeking social support reduced the effect of negative life events on psychological health concerns [100]. In addition, increasing social support also increases the tendency to use other adaptive cognitive emotion regulation strategies [145]. On the other hand, this may be due to culture. In societies with collectivist cultures such as Turkey, the identity of the individual is shaped within the social system to which he belongs, and individuals have strong social ties, social solidarity, and cooperation motives [146]. When an individual trusts the social support he has especially in a collectivist culture, he would be able to manage stress, overcome negative events, and would be happier. It is also suggested that by using other cognitive strategies such as positive reappraisal, people can tolerate or overcome negative life events more easily [78]. Individuals with high levels of positive appraisal have decreased depression and anxiety [77]. Individuals with a high level of optimism experience less psychological discomfort and are more satisfied with life despite negative life events [147]. Optimistic people try to make positive inferences from events when they encounter a difficult situation, they expect good things to happen, and as a result, they have a higher level of well-being than pessimists [142]. However, while optimism enables more active coping styles to be used, it is negatively associated with avoidance strategies and depression [148]. On the other hand, avoidance strategy has no effect on happiness in any group, and this finding is parallel with passive/disengaged coping strategy results [63]. An interesting finding of the study is that blaming others in order to protect their happiness level seems to be effective in individuals experiencing negative events. Moreover; the distinct difference between submissive and avoidance strategies in distinguishing between positive and negative-oriented coping profiles is also noteworthy.

Another finding of the study is that, according to groups there are different coping strategies necessary to reach happiness. Because when the perspective of negative events changes, the strategy of coping can also change, so the happiness can change. Individuals’ set points are not hedonic neutral. People may have different set points according to their nature. Even a person can have more than one happiness set point. Different components of well-being, such as positive emotions, negative emotions, and life satisfaction, can move in different directions. Individuals' adaptations to events vary; some individuals have the capacity to change their own set points. All these findings provide evidence that happiness level and set point can change [42]. At first, we developed the groups according to coping strategies and negative life events, then we focused on the coping strategies that have the highest level of happiness. And it is important to say that, there are different ways to go happiness for different groups. Many studies search for different coping strategies’ positive results or life events’ conclusions, but this research provides a clearer explanation of happiness. One of the important features that distinguishes this study from others is that, after the latent profile and latent class analyses in this study, meaningful subgroups were created, compared, and significant differences were revealed.

Positive-oriented coping strategy-less negative life events group scored higher on happiness than other groups and the negative-oriented coping strategy-more negative life events group scored the lowest happiness level. When the literature is examined, the findings of this study coincide with the results of previous studies [27, 41, 50, 51].

### Implications

Based on our data, it is indicated that adopting positive-oriented coping strategies may provide a highly promising route to happiness, regardless of whether life conditions are favorable or unfavorable (good or bad). Individuals especially young ones can think that their life is difficult, and current studies show that negative life events decrease well-being. However, we found that they can overcome these negative experiences with positive-oriented coping strategies. More specifically, it is possible to maintain the level of happiness in the face of negative events with an optimistic, seeking social support and positive reappraisal strategy respectively. Activities that transform young people into a change of positive-oriented perspective can be produced. At this point, activities to produce open-ended scenarios and to produce different and inventive solutions to these scenarios with the groups they interact with can be set up. In addition, it should not be forgotten that trainers are an important authority for social support in the cultural context. Increasing the mentoring service in an individual context and improving the trainers in this regard can be provided. Personal development awareness and training programs can be organized in the context of the findings of the research. So the difficulties of life are not an obstacle to being happy. However, while it is seen in the literature that young people mostly seek social support or follow avoidance strategies [24, 25, 35], the results of this study reveal that seeking social support positively affects happiness in different groups. This study also says that avoidance strategy acts like passive/disengaged coping strategies and does not affect happiness at the group level, while optimistic and positive reappraisal strategies are good strategies to maintain happiness. We believe these are potentially potent prescriptions to keep in mind. We would like to pay attention to a few important points here. For young people who have to struggle with difficulties and sometimes loneliness to lead a happier life, it is important to be reassuring while providing social support and to listen to their problems with interest and understanding [149]. Therefore, experts can help young people develop positive-oriented coping strategies to help them cope with their experiences in negative life events, perhaps to leave these negative experiences behind, and to increase their level of happiness. Increasing optimism, which emerges as one of the important strategies to maintain the happiness levels of young people, it will enable young people to be self-confident even under difficult conditions, to affirm their future expectations based on, to develop their sense of self [150], to evaluate their psychological resilience and to evaluate events in a positive way. In addition, it is recommended that almost everyone focus on the future and their goals to increase their own level of optimism.

### Limitations and future research

In light of the valuable constructions of the literature, the current study had some limitations. Although data were obtained from a large sample, they were self-reported, cross-sectional, and quantitative. Therefore, the findings were limited to the answers of the participants. However, the lack of inclusion and exclusion criteria for participant selection in the study may limit the generalizability of the results of the study and the capacity of the study sample to provide full representation. For the purpose of the study, only young people studying at different levels in the university were included in the research. However, because the study group does not have a balanced gender distribution and the question of determining the universities of the participants is not included in the research form, the inability to determine how many people from which universities participated in the research is a limitation. For this reason, determining the inclusion and exclusion criteria more clearly in future similar studies may contribute to supporting the findings of this study and help to obtain more comprehensive results. Another limitation of this study is the valuations were obtained retrospectively. This prevents the evaluation of individuals' previous and subsequent situations. Making longitudinal designs in future research may provide healthier results. In future research, both quantitative data and qualitative data obtained as a result of observation and interviews can be used together. In this way, it may be possible to obtain more detailed and realistic results. Future research could also examine the longitudinal situations of negative life event classes and coping strategy profiles by latent transition analysis and their effects on happiness. In addition, the effects of different coping strategies in different life conditions can be examined in detail.

## Conclusions

This study was the first to consider profiles according to coping strategies and negative life events together. After having profiles, we compare the happiness levels and explore a clearer picture for individuals. We found that “Happiness levels of those who have positive-oriented coping strategies do not change depending on negative life conditions.” If people use slightly positive-oriented coping strategies, their happiness levels may decrease when life conditions become more difficult. However, if people's coping strategies are positive-oriented, their happiness levels are high, and their happiness levels do not decrease significantly even if their life conditions become more difficult. The level of happiness is low in individuals with a negative-oriented coping strategy, and fewer or more negative life events do not change the level of happiness.

## Data Availability

The datasets generated during and/or analyzed during the current study are available from the corresponding author on reasonable request.
